# Using phosphate amendments to reduce bioaccessible Pb in contaminated soils: A meta-analysis

**DOI:** 10.3389/fsoil.2022.1028328

**Published:** 2022-11-07

**Authors:** Manfred M. Mayer, Nicholas T. Basta, Kirk G. Scheckel

**Affiliations:** 1 School of Environment and Natural Resources, The Ohio State University, Columbus, OH, United States,; 2 U.S. Environmental Protection Agency, Center for Environmental Solutions and Emergency Response, Land Remediation and Technology Division, Cincinnati, OH, United States

**Keywords:** lead (Pb), phosphorous (P), remediation, bioaccessibility, soil

## Abstract

Measuring the reduction of *in vitro* bioaccessible (IVBA) Pb from the addition of phosphate amendments has been researched for more than 20 years. A range of effects have been observed from increases in IVBA Pb to almost 100% reduction. This study determined the mean change in IVBA Pb as a fraction of total Pb (AC) and relative to the IVBA Pb of the control soil (RC) with a random effects meta-analysis. Forty-four studies that investigated the ability of inorganic phosphate amendments to reduce IVBA Pb were identified through 5 databases. These studies were split into 3 groups: primary, secondary, and EPA Method 1340 based on selection criteria, with the primary group being utilized for subgroup analysis and meta-regression. The mean AC was approximately −12% and mean RC was approximately −25% for the primary and secondary groups. For the EPA Method 1340 group, the mean AC was −5% and mean RC was −8%. The results of subgroup analysis identified the phosphorous amendment applied and contamination source as having a significant effect on the AC and RC. Soluble amendments reduce bioaccessible Pb more than insoluble amendments and phosphoric acid is more effective than other phosphate amendments. Urban Pb contamination associated with legacy Pb-paint and tetraethyl Pb from gasoline showed lower reductions than other sources such as shooting ranges and smelting operations. Meta-regression identified high IVBA Pb in the control, low incubated soil pH, and high total Pb with the greater reductions in AC and RC. In order to facilitate comparisons across future remediation research, a set of minimum reported data should be included in published studies and researchers should use standardized *in vitro* bioaccessibility methods developed for P-treated soils. Additionally, a shared data repository should be created for soil remediation research to enhance available soil property information and better identify unique materials.

## 1 Introduction

Lead (Pb) is a widespread contaminant with the World Health Organization classifying it as one of ten chemicals of major public concern (1). Exposure to Pb leads to Pb poisoning, which can cause behavioral problems and learning deficiencies in children, and has negative impacts on almost every function in the body, regardless of age (2). Currently 3.5 μg Pb dL^−1^ is the reference value to identify dangerous levels of blood Pb in children in the U.S. (3) Low levels of Pb are potentially harmful, with the Center for Disease Control and Prevention stating there is no safe amount of detectable Pb in a child’s blood (4).

Ingestion of contaminated soil is the most common exposure pathway leading to elevated blood Pb (4). Once Pb is deposited in the soil, it does not leach further down the soil profile, nor will it be translocated except by erosion processes (5). This makes Pb a persistent contaminant in the soil, requiring intervention. The U.S. Environmental Protection Agency (U.S. EPA) regulates total Pb in soil; the current limits are 400 ppm Pb in children’s play areas and 1200 ppm Pb in other soils (6). Total Pb is reduced through techniques such as excavation and removal, soil washing, or physical stabilization. These processes are expensive, destructive, and may not be feasible for small sites or difficult to access areas (7, 8).

The issues associated with reducing total Pb have led to research of on site (*in situ*) remediation techniques. *In situ* remediation focuses on reducing the fraction of Pb that will be mobilized by an organism if exposed, termed bioavailable Pb. The addition of phosphate amendments to Pb contaminated soil to reduce bioavailable has been researched since 1993 (9). Theoretically, the addition of phosphate amendments should form Pb-phosphate minerals from almost any form of Pb that may be present in the soil. Pb-phosphates, especially pyromorphite-like minerals, are stable and insoluble, even under stomach-like conditions (10).

The ideal way to measure changes in bioavailable Pb is through animal dosing (*in vivo*) studies, as it is a direct measurement of the amount of Pb mobilized. Yet, animal dosing trials are expensive, require long periods of time to complete, and availability is extremely limited. This has led to the development of laboratory (*in vitro*) methods designed to replicate the digestive processes of *in vivo* studies. While *in vitro* methods are not direct measurements of bioavailability, they have been found to be highly correlated with *in vivo* studies (11–14). In order to differentiate between *in vivo* and *in vitro* measurements, *in vitro* results are referred to as bioaccessible.

Bench and field studies have reported changes in bioaccessible Pb from the addition of phosphate amendments for the past 2 decades. Some studies report no reduction in bioaccessible Pb (15, 16) while others have reported over 60% reductions in Pb bioaccessibility (17, 18). The effectiveness of phosphate treatments is unclear, and the relationship between the addition of phosphate and formation of chloropyromorphite is complex (9). The objectives of this study are to **1)** determine the average reduction in bioaccessible Pb from the addition of phosphate amendments and **2)** determine factors affecting the reduction of bioaccessible Pb by the addition of phosphate amendments.

## 2 Methods

### 2.1 Candidate identification

All literature searches were conducted following the guidelines in the Preferred Reporting Items for Systematic Reviews and Meta-Analyses statement and checklist (item numbers 5 through 8) (19).

#### 2.1.1 Search strategy and databases

The search phrase used was (“lead” or “pb” or “metal?”) and “phos*” and (“pbet” or “physiologically based extraction test” or “bioaccessib*” or “sbrc” or “1340” or “rbalp” or “ubm” or “rivm” or “sbet”). Searches were not case sensitive. The first set parenthesis returned all articles with the words “Pb”, “lead”, metal, or metals. The next terms filtered the results to only include articles with words such as phosphorous, phosphoric or phosphate. The second set of parentheses was the final filter and contained methods designed to replicate the digestive system and the word bioaccessible. Inclusion of the term “glycine” in the second set of parentheses was explored but it greatly increased the number of results in the search without generating any additional relevant hits.

In order to increase the number of results and reduce bias, five databases were used. The five databases used are as follows: Web of Science (Clarivate Analytics, Philadelphia, Pennsylvania, US), GeoRef (American Geosciences Institute, Alexandria, Virginia, US), Scopus (Elsevier, Amsterdam, Netherlands), ProQuest Environmental Science Index and ProQuest Dissertations and Theses (ProQuest, Ann Arbor, Michigan, US). The above search phrase was entered exactly as written in Web of Science, GeoRef, and Scopus. The above phrase was modified by adding “noft” prior to the first parenthesis for ProQuest databases. This restricted the search to titles, abstracts, and key words. All searches were conducted on April 23^rd^, 2021. Additionally, the references of Scheckel et al. (9) were searched for possible candidates.

#### 2.1.2 Screening

All duplicate results were removed. Any study title that was clearly not relevant to this meta-analysis was removed. Abstracts were screened next. Studies were removed if they did not investigate a soil remediated with inorganic phosphate amendments (P amendments) or if bioaccessible Pb was not measured. Additionally, studies that only use sequential extraction methods were removed. While sequential extraction studies occasionally use the term bioaccessible, all results are for operationally defined speciation, meaning the extracting agents define the type of Pb removed (20). There is evidence that the extraction procedures themselves can induce chemical changes in the sample and misrepresent the fractions an author may have deemed bioaccessible (21).

#### 2.1.3 Eligibility

Studies were separated into three groups: primary, secondary, and EPA Method 1340. For all categories, studies must have 1) used a method to measure bioaccessible Pb meant to approximate a human digestive system and 2) reported a measure of gastric phase bioaccessible Pb in the treated soil. The bioaccessibility of the gastric phase shows greater correlation than the intestinal phase for *in vivo in vitro* correlations and is more frequently reported. Specific treatments within a study or whole studies were excluded if another soil amendment, such as compost or iron oxide, is added in addition to the P amendment. Specific treatments within a study or whole studies were excluded if the incubation was conducted with non-environmentally relevant conditions. This included maintaining high soil temperature or soil moisture significantly greater than the water holding capacity. Studies that added a liming agent after the addition of P amendment were not excluded. Studies which had a control IVBA percentage greater than 120% were excluded.

Soils in the primary group were used for meta-regression and subgroup analysis. In addition to the criteria above minimum eligibility requirements for the primary analysis were 1) reported total Pb, 2) the amount of phosphorous (P) added 3) bioaccessible Pb of the control, 4) bioaccessible Pb in the treated soil, 5) pH of the control soil, and 6) an incubated pH of the treated soil, without the addition of lime. Soil pH was included in the criteria because it is an important control on the solubility of Pb in soil (22).

Field scale studies were excluded from primary group due to the large number of confounding environmental variables, such as wetting and drying cycles, lack of temperature control, and spatial heterogeneity. Other studies that investigated the effect of P amendments on bioaccessible Pb but did not meet the eligibility requirements of the primary group were included in the secondary group. Studies that used EPA Method 1340 (0.4 M glycine at pH 1.5) (23) to measure bioaccessible Pb were separated into a third group as this extraction is not suitable for phosphate-amended soils (24). The Solubility Bioaccessibility Research Consortium method is an equivalent method to EPA Method 1340 (25).

### 2.2 Data extraction

#### 2.2.1 Quantitative

All data were extracted manually from the texts and recorded in Excel (Microsoft, Redmond, Washington, US). Data reported in tables were recorded in Excel. Data that were only presented in graphs was extracted with the aid of the “metaDigitise” package (26) for R (R Foundation for Statistical Computing, Vienna, Austria). To enhance the accuracy of the point and click interface of the “metaDigitise” package, a magnifier (Microsoft, Redmond, Washington, US) was used at 300% magnification. Bioaccessible Pb in the control soil and bioaccessible Pb in the treated soil were recorded for all studies. Total Pb, P added, pH of the control, and incubated, unlimed pH of the treated soil were recorded for soils included in the primary analysis. Additional quantitative soil properties beyond the minimum criteria, such as organic carbon percentage, clay percentage, and cation exchange capacity, were recorded for soils in the primary group if available.

##### 2.2.1.1 Missing variance data

Not all studies reported variance data for bioaccessible Pb. Corresponding authors of papers included in the primary analysis were contacted for the missing variance data. If the author did not respond, a simple imputation of the missing standard deviation was used based on Furukawa et al. (27). This method imputes missing standard deviation as the pooled standard deviation of all treatments which reported a standard deviation. A separate imputed standard deviation was found for control soils and for treated soils based on soils in that were included in the primary group (Equation 1).

Equation 1. Calculation of pooled standard deviation. Excerpted from Furukawa et al. (27).


$${\text{SD}}_{\text{pooled }}=\sqrt{\frac{\sum \left(n_{i}-1\right)⋆SD_{i}^{2}}{\sum \left(n_{i}-1\right)}}$$

n_*i*_ = number of samples of the *i*th study

SD_*i*_ = Standard deviation of the *i*th study

The calculated values from the primary analysis were used as the imputed standard deviation for all three groups. For field studies, a set value of 10% was used if the standard deviation was missing, as these are more variable than laboratory studies.

#### 2.2.2 Qualitative

The specific P amendment added was recorded as it may influence the effectiveness of the treatment. Additional information recorded include geographic area, contamination source, and bioaccessibility extraction method used.

### 2.3 Statistics

#### Random effects model

Fixed effect and random effect models are the two most common ways to analyze meta-analysis data. The fixed effect model is only appropriate if the studies are assumed to be drawing from one population with one common effect size (28). Since soils are very heterogenous, it is unlikely that this assumption is valid. Therefore, the random effects model was used. Within this model, studies are weighted by inverse variance. The random effects model estimates a true mean treatment effect from a distribution of treatment effects, since it is assumed that more than one population is present. The Hartung-Knapp-Sidik-Jonkman method was used (29). In order to incorporate the multiple possible populations, a between-study variance estimated was needed prior to estimation of the true mean treatment effect. Based on the review by Veroniki et al. (30), the restricted maximum likelihood was used because the data in this analysis are continuous.

#### 2.3.2 Measures of treatment effects

Two different treatment effects were investigated in this meta-analysis: Absolute change in *in vitro* bioaccessible (IVBA) Pb (AC) and relative change in IVBA Pb (RC). AC is the percentage of IVBA Pb changed in relation to total Pb (Equation 1 and 2)

Equation 1. IVBA Pb percentage.


$$\text{ IVBA Pb}(\%)=\frac{\text{ bioaccessible Pb}\left(\frac{\text{mg}}{\text{kg}}\right)}{\text{ Total Pb}\left(\frac{\text{mg}}{\text{kg}}\right)}$$

Equation 2. Absolute change in IVBA Pb (AC).


$$\begin{array}{l} \text{\% absolute change in IVBA Pb }\left(\text{AC}\right)\\ \text{= treatment IVBA Pb}\left(\%\right)\;-\;\text{control IVBA Pb }\left(\%\right) \end{array}$$

RC is the change in IVBA Pb as a fraction of IVBA Pb in the control soil (Equation 3).

Equation 3. Relative change in IVBA Pb (RC).


$$\begin{array}{l} \%\text{ relative change in IVBA Pb }(\text{RC})\\ =\left(\frac{\text{AC}}{\text{ control IVBA Pb }(\%)}\right)*100\% \end{array}$$

RC is analogous to treatment effect ratio (TER) minus 1. RC was used as Scheckel et al. (9) used TER to compare results across studies.

Treatment effects were analyzed as unstandardized mean differences between the control and treated samples. Standard deviations underwent the same transformations as the means when converting from AC to RC.

#### 2.3.3 Unit of analysis

Many studies compare multiple treatments to one set of control samples. In order to appropriately weight each sample and minimize counting control samples multiple times, a composite sample was formed for all non-control treatments in a given soil (31). The composite samples were the units of analysis for purposes of the determination of the summary treatment effects described above. A composite sample is referred to as a soil. Treatment means were combined using a weighted average (weighted by number of samples). Standard deviations were pooled appropriately for laboratory studies (Equation 4).

Equation 4. Pooling of standard deviations.


$${\text{SD}}_{\text{pooled }}=\sqrt{\frac{\sum \left(n_{i}-1\right)⋆SD_{i}^{2}}{\sum \left(n_{i}-1\right)}}$$

SD_*i*_ = Standard deviation of the *i*th treatment

n_*i*_ = number samples of treatment of the *i*th treatment

These composite samples suffer from information loss, specifically of qualitative parameters.

#### 2.3.4 Subgroup analysis and meta-regression

Subgroup analysis was performed for qualitative variables using a fully random effects model. To accomplish subgroup analysis, composite samples as described above were formed for each soil/qualitative parameter pairing. While this does lead to multiple counting of some control samples, it was determined to be less egregious than using individual treatments. Contamination source was split into 4 categories: mining and smelting, shooting ranges, urban (Pb-based paint or leaded gasoline), and other. Amendment type was defined as soluble or insoluble. If a single treatment included an insoluble and soluble P amendment, it was identified as soluble. The influence of the P amendment was also investigated as added phosphoric acid or did not add phosphoric acid (referred to as acid factor).

Meta-regression of treatment effects was done by continuous variables including control IVBA Pb, year, total Pb, P added, P: Pb molar ratio, control soil pH, incubated soil pH, organic C percentage, CEC, incubation time, and publication year. Meta-regression utilized the composite samples formed for the determination of the mean treatment effects. All continuous variables were averaged appropriately. Significant relationships were determined using single regression.

#### 2.3.5 Outliers

Outliers were identified as soils with 95% confidence intervals which do not overlap with the 95% confidence interval of the pooled average (32).

#### 2.3.6 Software

Data transformation was conducted on extracted data with Excel. All analysis was conducted in R using the “dmetar” package and accompanying guide “Doing meta-analysis in R” (32). This guide also used the packages “meta” (33) and “metafor” (34).

## 3 Results

### 3.1 Eligible literature

**FIGURE 1:**
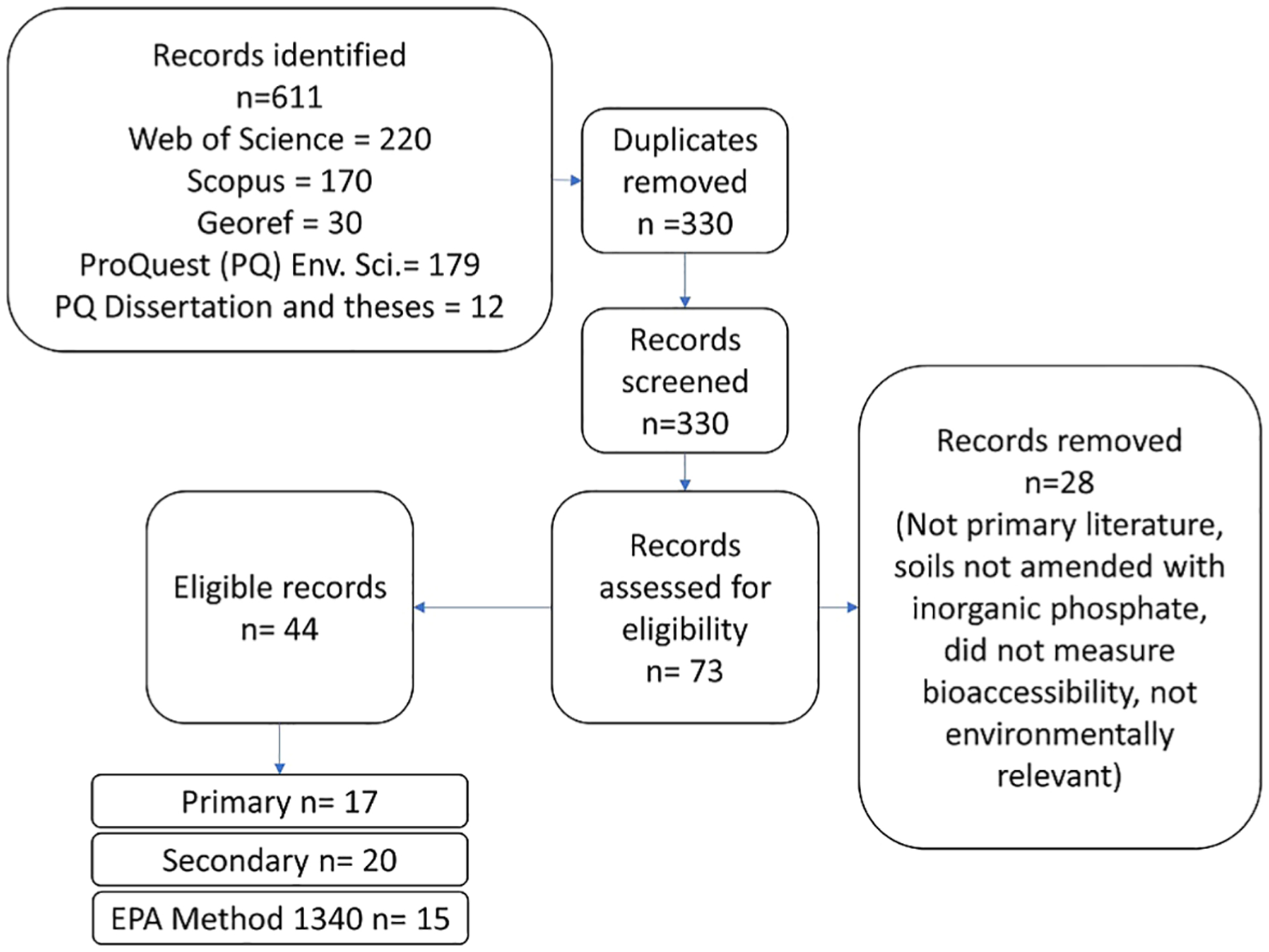
Flowchart of literature search and eligible records in this meta-analysis.

Searching 5 databases returned 611 hits and 330 unique results. After initial title, abstract screening, and comparison against eligibility criteria 44 candidates remained. Literature identified was published between 2000 and 2020. After comparison to the criteria above, 17 articles were included in the primary group, 20 articles were included in the secondary group, and 15 articles were included in the EPA Method 1340 group (Figure 1).

Articles were included in multiple groups when necessary. For example, Beyer et al. (35) reports 2 different P-amended soils. One of the soils was limed and only reported a limed incubated pH while the other soil was not limed. Therefore, the non-limed soil was included in the primary analysis and the limed soil was included in the secondary group. The authors used EPA Method 1340 in addition to other bioaccessibility methods, so data from the soils investigated by Beyer et al. (35) were also included in the EPA Method 1340 group.

The 17 articles in the primary group represent a total of 40 soils with 142 treatments and 568 samples (Table 1).

The 20 articles in the secondary group represent 46 soils with 186 treatments and 529 samples. The 15 articles in the EPA Method 1340 group represent 38 soils with 169 treatments and 395 samples. These articles can be found in Table S1.

#### 3.1.1 Missing variance data

The imputed pooled standard deviation for control soils was4.2% based on 37 reported control soils. The imputed pooled standard deviation for treated soil was 3.2% based on 120 treatments which reported standard deviation. Six soils in the primary group, 18 soils in the secondary group, and 10 soils in the EPA Method 1340 group used an imputed standard deviation.

### 3.2 Treatment effects

#### 3.2.1 Distribution of treatment effects

**FIGURE 2:**
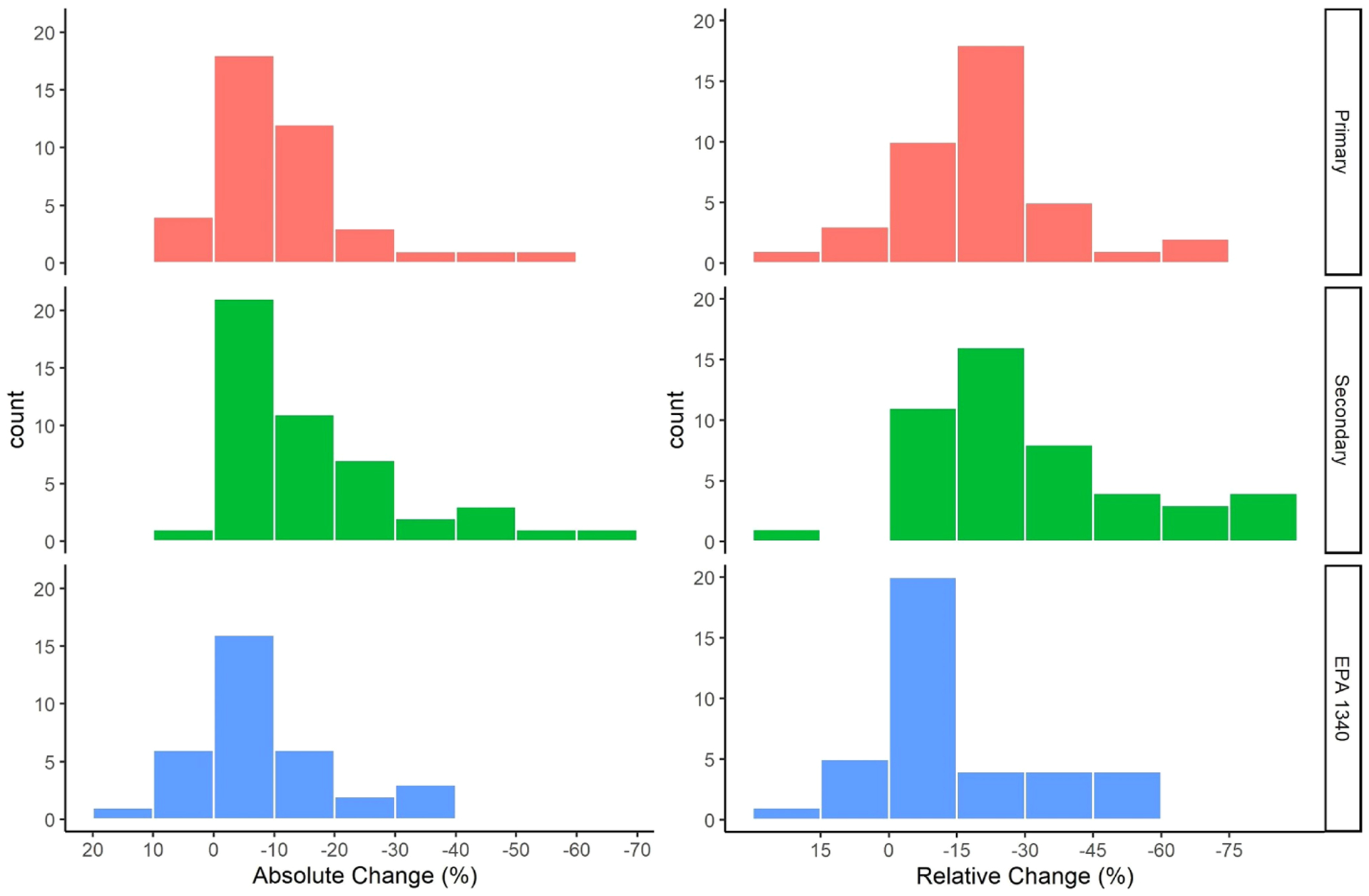
Distribution of relative change in IVBA Pb (%) for soils in each analysis group.

A positive AC or RC is an increase in the bioaccessibility of Pb while a negative AC or RC is a reduction in the bioaccessibility of Pb. The range of AC observed in individual treatments was 21% to −84% while the range of RC was 50% to −98%. The AC observed for soils ranged from 8% to − 52%, 6% to −66%, and 13% to −34% for the primary, secondary, and EPA Method 1340 groups respectively. The greatest number of soils had an AC between 0% and −10% for all groups. The RC observed for soils ranged from a 16% to −72%, 15% to −82%, and 17% to −59% for soils in the primary, secondary, and EPA Method 1340 groups respectively. The greatest number of soils had a RC between −15 and −30% for the primary and secondary groups and between 0 and −15% for EPA Method 1340 (Figure 2).

#### 3.2.2 Average treatment effect

Ten soils in the secondary group and 11 soils in the EPA Method 1340 group could not be used for determination of the summary treatment effects as there appeared to be a single control sample for those soils. Four soils in the EPA Method 1340 group could not be used for determination of the summary treatment effects as the control IVBA Pb was not reported.

The number of studies included, mean treatment effect, 95% confidence interval (CI) of the mean treatment effect, p-value of the treatment effect, percentage of real differences between studies (I^2^), and the square root of the between study variance estimator (τ) for AC and RC for all three analysis groups are reported in Table 2.

The estimate of the summary effect for all groups and treatment effects are reported as average percent change [lower bound of 95% CI, upper bound of 95% CI] (ex. an average reduction of 5% with a 95% CI ranging from 15% reduction to a 5% increase would be expressed as −5%[−15%,5%]). For the primary group (n=40), the mean AC was −11.4% [−15.4%,−7.4%] and mean RC −21.4% [−26.7%,−16.1%]. For the secondary group (n=36), the mean AC was −15.6% [−21.4%,−9.7%] and mean RC was −32.3% [−40.5%,−24.2%]. AC and RC were determined for the secondary group without field studies (n=29); the mean AC was −12.4% [−18.7%, −6.1%] and the mean RC was −27.2% [−35.4%, −18.9%]. The mean AC and RC for the field studies in the secondary group was −28.5% [−41.8%, −15.2%] and −53.6% [−74.4%, −32.9%], respectively. For the EPA Method 1340 group (n=23), the mean AC −5.4% [−10.4%,−0.4%] and mean RC was −8.3%[−16.1%,−0.6%]. With the exception of field studies, all groups had an I^2^ greater than 94%. The between study standard deviation ranged from 11% to 16% for AC and 15% to 23% for RC. The addition of P amendments showed a significant reduction (p< 0.5) for all groups and treatment effects.

#### 3.2.3 Subgroup analysis and meta-regression

**FIGURE 3:**
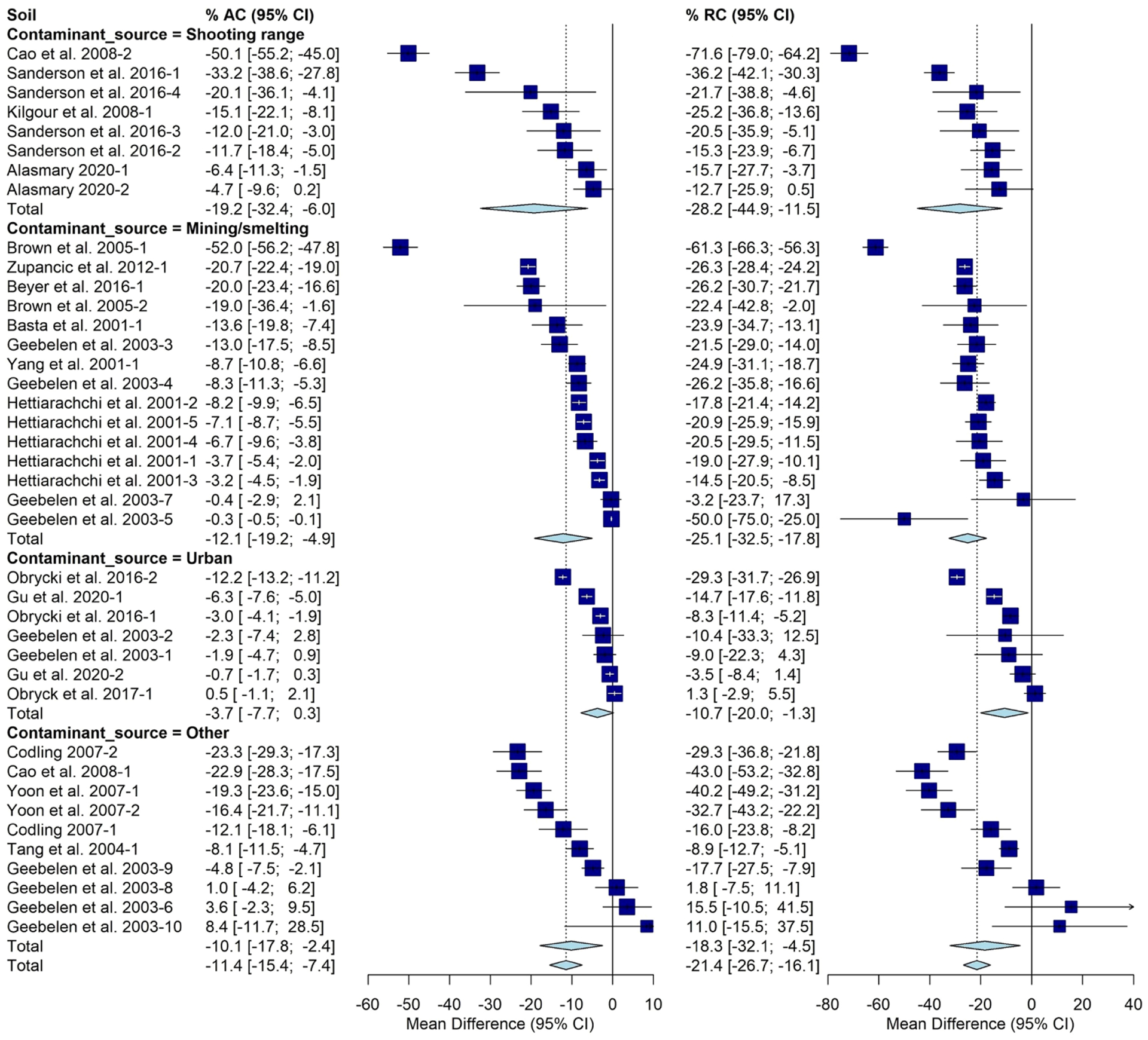
Fully random effects forest plot for AC and RC grouped by contaminant source. Squares represent the mean difference for a soil and error bars are 95% CI. Diamonds represent the 95% CI of the mean difference between the control and treated soil for each subgroup and the overall treatment effect.

The AC varied significantly based on contaminant source with an average of −19% [−32%, −6%] determined for shooting ranges. Mining/smelting sources averaged −12% [−19%, −5%].Urban sources averaged −4% [−8%,0%] and all other sources had a −10% [−18%, −2%] change. Significant differences were observed with respect to contaminant source for RC as well. The average was −28% [−45%, −11%] for shooting ranges, −25% [−33%, −18%] for mining and smelting Pb sources, −11% [−20%, −1%] for urban Pb sources, and −18%[−32%,−5%] for other Pb sources (Figure 3).

**FIGURE 4:**
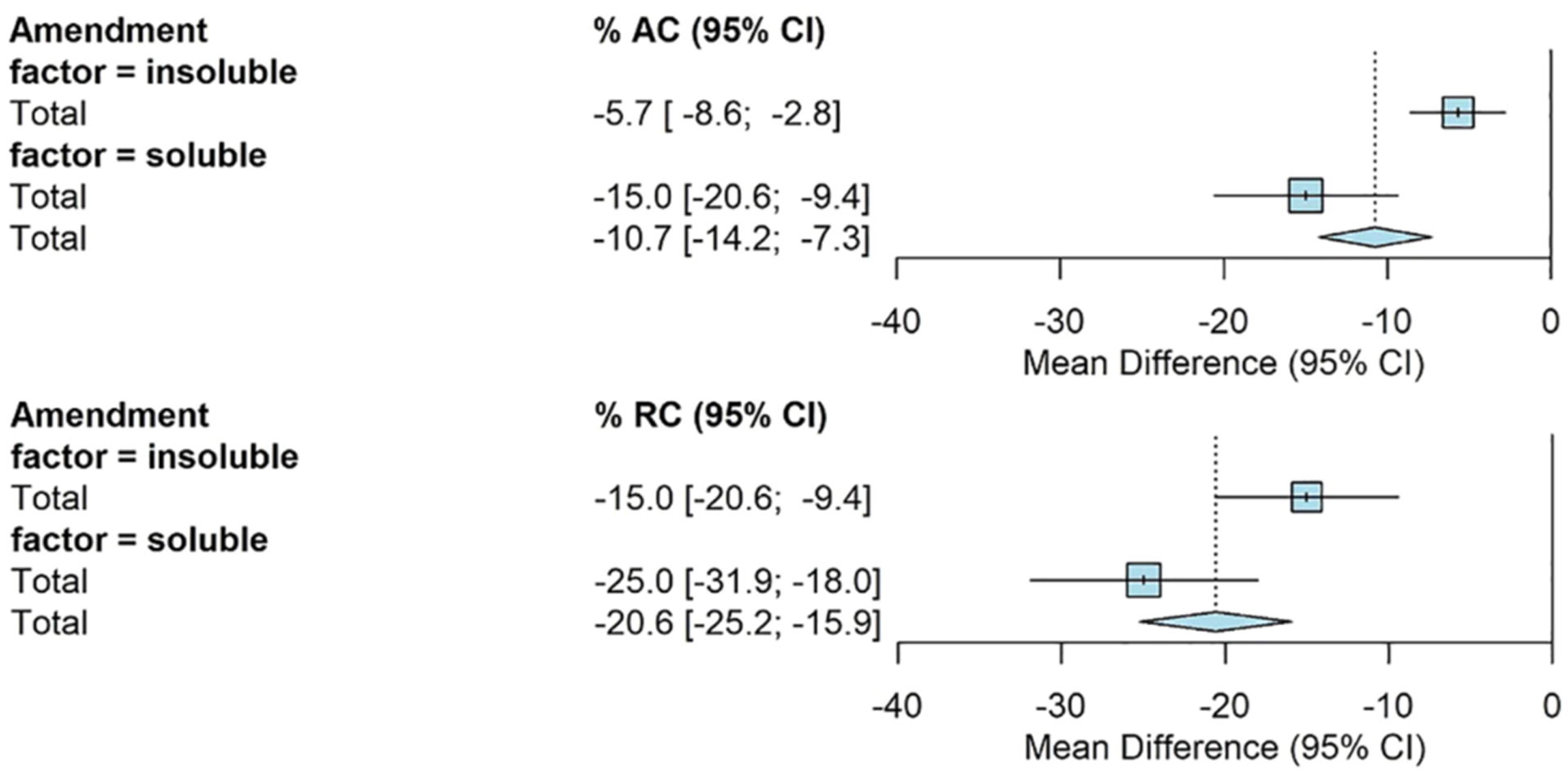
Fully random effects forest plot for AC and RC by amendment type. Squares represent average treatment effect for each subgroup with error bars denoting the 95% CI. Diamonds represent the 95% CI of the overall treatment effects.

Amendment type had a significant effect on AC and RC. The mean AC for insoluble amendments was −6% [−9%, −3%] and −15% [−20%, −9%] for soluble amendments. The average RC when insoluble amendments were applied was −15% [−21%, −9%] compared to −25% [−32%, −18%] when soluble amendments were applied (Figure 4).

**FIGURE 5:**
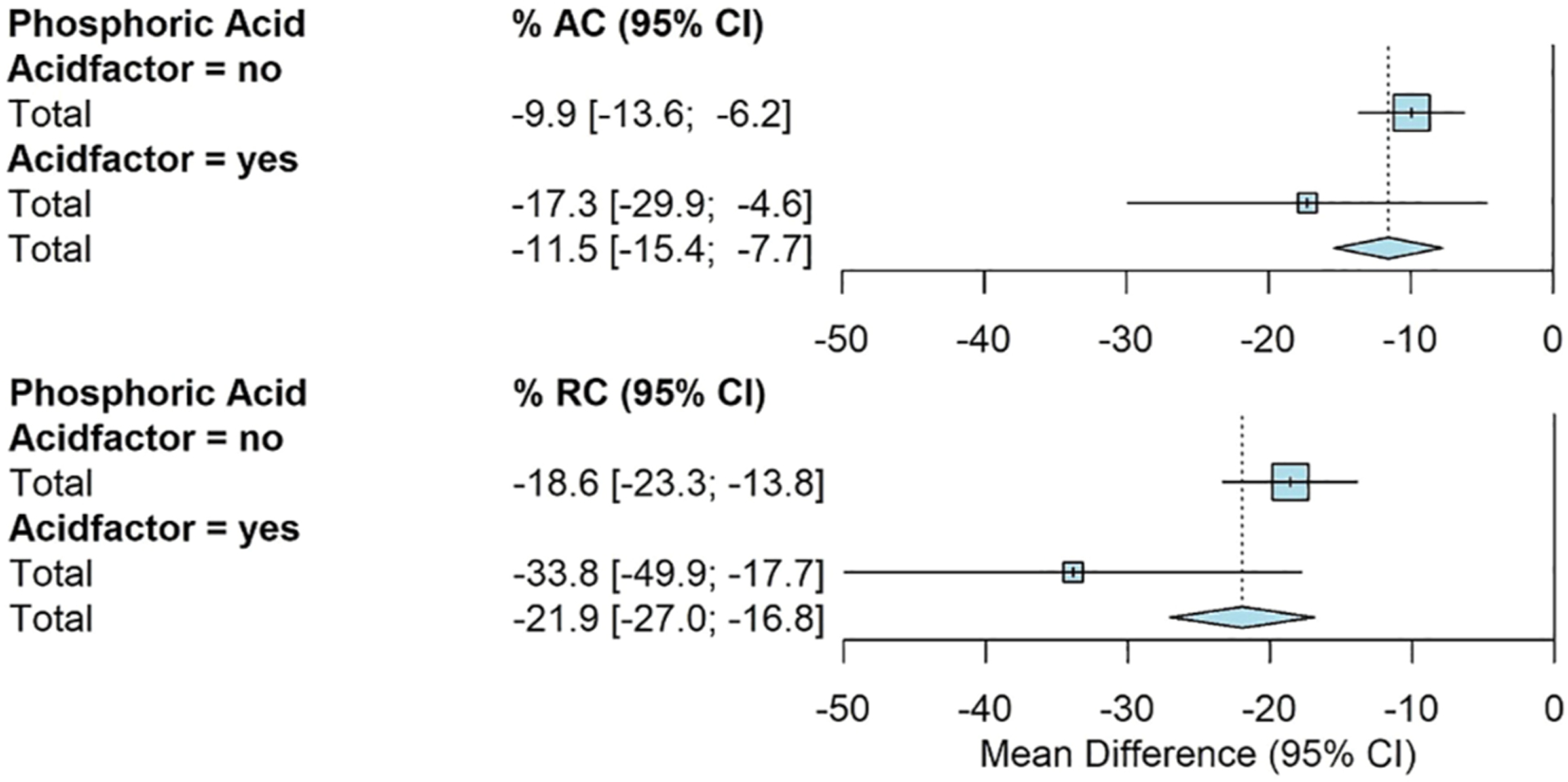
Fully random effects forest plot for AC and RC by acid factor. Squares represent average treatment effect for each subgroup with error bars denoting the 95% CI. Diamonds represent the 95% CI of the overall treatment effects.

The use of phosphoric acid showed significantly greater reductions for RC. The addition of phosphoric acid had an average RC of −34% [−50%, −18%] compared to −19% [−23%, −14%] for all other amendments (Figure 5).

The use of different composite samples for subgroup analysis, formed as soil/qualitative parameter pairing, did not have a pronounced effect on the overall treatment effects. The averages and 95% CIs were within 1% of the values determined using the composite for a soil as the unit of analysis.

**FIGURE 6:**
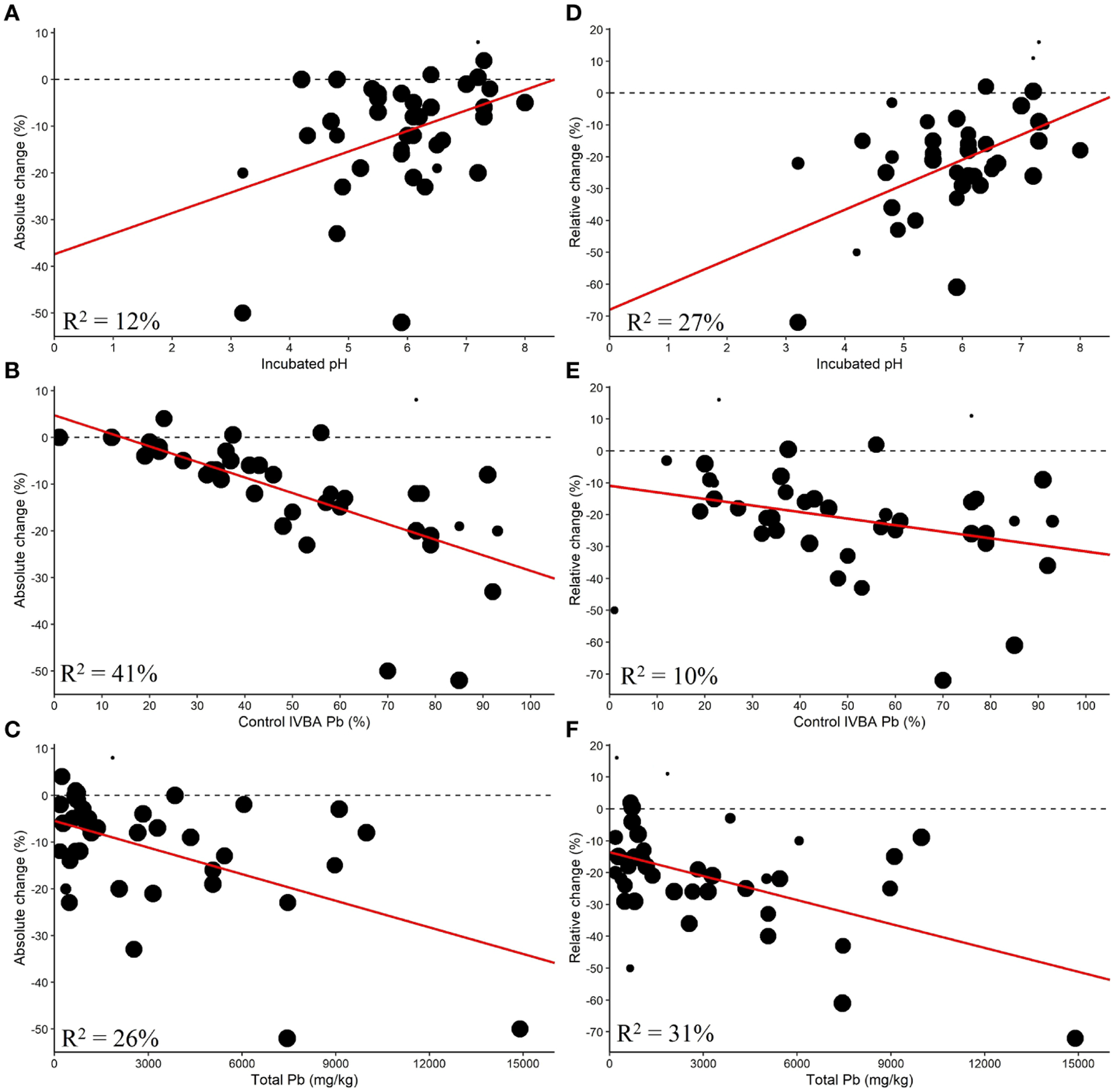
Single linear meta-regression for AC by **(A)** incubated pH, **(B)** control IVBA Pb, and **(C)** total Pb and RC by **(D)** incubated pH, **(E)** control IVBA Pb, and **(F)** total Pb.

The control IVBA Pb (%), incubated pH, and total Pb were found to be significantly correlated with AC (Figure 6).

Control IVBA Pb explained 46% of the difference between studies, but the data were much more variable for soil with greater control IVBA Pb. The control IVBA percentage may influence RC as it had a p-value less than 0.1. Higher control IVBA was correlated with greater reductions in AC and RC. Incubated pH had an R^2^ of 12% with respect to AC. For RC, Incubated pH explained 27% of the differences between studies. Lower incubated soil pH corresponded with greater reductions in AC and RC. Total Pb was found to be significantly correlated with AC and RC, with higher total Pb being associated with greater reductions in AC and RC. This relationship may be contingent on the fewer studies at high levels of total Pb.

Amount of P added, control soil pH, P:Pb molar ratio, organic C percentage (n =38), cation exchange capacity (n=26), clay percentage (n=13), incubation time (n=38), and year of publication were not significantly correlated with AC or RC.

#### 3.2.4 Outliers

A large number of outliers (n ≥ 7) were observed for all groups. This is due to the definition of an outlier in this context and the wide range of effects reported in literature. No outliers were removed as there was no indication any soil was treated or incubated in a markedly different manner.

## 4 Discussion

### 4.1 Analysis groups

All groups had high I^2^ values. This indicates that the majority of the differences between soils arise from real differences between them as opposed to sampling error. The high observed heterogeneity is attributable to the high precision (low standard deviation) associated with the laboratory nature of the majority of the studies, the large number of soils, and wide range of reductions observed.

The visual differences in the distribution of treatment effects (Figure 2) for the primary and secondary groups can be attributed to the inclusion of field studies. The inclusion of field studies also led to greater average reductions for AC and RC in the secondary group. Field studies overrepresented a single location as 5 out the 8 soils from field studies are in Jasper County, MO (48–52). Additionally, 4 of these studies only applied phosphoric acid and at least two studies reported data for the same soil in different years. The two soils investigated in Brown et al. (53) are located in Tar Creek, OK, which borders Jasper County, MO. The greater reductions for AC and RC observed for field studies should not be considered as a relevant conclusion due to the limited geographic area represented and the use of phosphoric acid in half the reported soils.

The EPA Method 1340 group had a lower average AC and RC than the primary and secondary groups. The implication of this is discussed further in section 4.5: Recommendations.

### 4.2 Significant findings

The results of this analysis identified amendment type and incubated pH as having significant effects for both AC and RC. There are direct relationships between these factors as phosphoric acid and TSP both lower pH and are soluble amendments. The addition of phosphoric acid, which showed significantly greater reductions from other amendments in this analysis, lowered soil pH to between 3 and 6 in various studies (18, 37, 38, 41, 43). Hettiarachchi et al. (37) and Obrycki et al. (16) investigated the effects of soil acidification on the ability of P amendments to reduce IVBA Pb. The soils reached approximately pH 5.5, but no significant differences were observed compared to non-acidified treatments. Zhang and Ryan (54) reported the formation of chloropyromorphite in solution was reduced at pH 6 and above due to incomplete mineral dissolution. Lower soil pH increases the dissolution of Pb minerals (45), potentially explaining the inverse relationship between incubated soil pH and RC. Further, research needs to be conducted to clarify the role of soil pH without the influence of differing P amendments.

Urban contaminant sources showed lower reductions for both AC and RC. There are fewer sites that have investigated urban soils than all other sources, and all soils were above pH 6. With the exception of one of the soils in Geebelen et al. (15) all urban sites had a total Pb below 1300 mg kg^−1^. Low total Pb and near neutral to slightly alkaline pH may explain a portion of the observed difference. Another potential influence is the relatively few study locations observed as 5 out the 7 urban soils are from the Great Lakes region, with 3 soils from Cleveland, OH and 2 from Chicago, IL. Further research is needed to identify if the lower reductions observed are representative of urban contamination sources.

### 4.3 Lack of significance for other variables

No significance was found for P: Pb molar ratio or the amount of P added across studies. A number of studies report greater reductions with increasing P added (37, 38, 42, 44, 53, 55, 56). The lack of relationship observed across soils in this analysis suggests that the reduction in bioaccessible Pb by P amendments is a function of the Pb-mineral phase or soil properties and not P:Pb ratio.

No significant relationship was found for organic C percentage for AC or RC. High dissolved organic matter has been shown to increase Pb solubility at high pH (57). Organic matter has also been shown to inhibit Pb-phosphate mineral formation in solution at low pH (58). Yamada and Katoh (59) found dissolved organic matter to complex with Pb in solution despite the presence of an excess of phosphate. The true relationship that exists between organic matter, reduction of bioaccessible Pb in soil and the addition of P amendments may be too complex to capture through the observational nature of meta-analysis.

The majority of studies in this analysis investigated a single soil or a small number of soils from a single geographic region. The influence of different P amendments, limited reported soil properties, and variety of methods employed diminished the ability of this study to determine the effect of soil properties. Studies have found significant correlations between bioaccessible Pb and soil properties (60) but this has not been researched specifically for soils remediated with P amendments. Further primary research is needed to ascertain the influence of soil properties on the ability of P to reduce bioaccessible soil Pb.

### 4.4 Limitations and assumptions

There were a variety of ways in which data had to be manipulated to extract the information of interest. For example, P added in Gu et al. (46) had to be calculated from a 4:1 molar ratio of P: [Cd + Zn + Pb] using information provided. The calculated value assumed that the authors did not markedly increase or decrease the amount of P added by applying easier to measure amount of amendment. Additionally, for some soils, treated IVBA Pb had to be back calculated from TER and control IVBA percentage. Due to assumptions needed to manipulate data and error due to rounding the possibility of a soil being slightly misrepresented exists.

Within the primary group, there were 4 different extraction methods used at differing pHs leading to a total of 9 different extraction method/pH combinations. The pH and extractant both influence the amount of bioaccessible Pb measured. Obrycki et al. (44) Scheckel et al. (49), and Moseley et al. (56) found that bioaccessibility of Pb decreased as extraction pH increased. Additionally, the relationships between different extraction methods are unclear (14, 61). The variety of bioaccessibility methods employed made it impossible to attempt comparison with subgroup analysis. Because the different methods were attempting to measure the same fraction of Pb (i.e. bioaccessible Pb), they were treated as comparable for the purposes of this analysis. It is worth noting that if a reduction in IVBA Pb occurred for one extraction method, it generally occurred in other extraction methods. Assumptions about the comparability of methods were also needed for soil pH and organic C. The soil: solution ratio varied from 1:1 to 1:5 for pH measurement and a number of different methods were used for determination of organic C.

**FIGURE 7:**
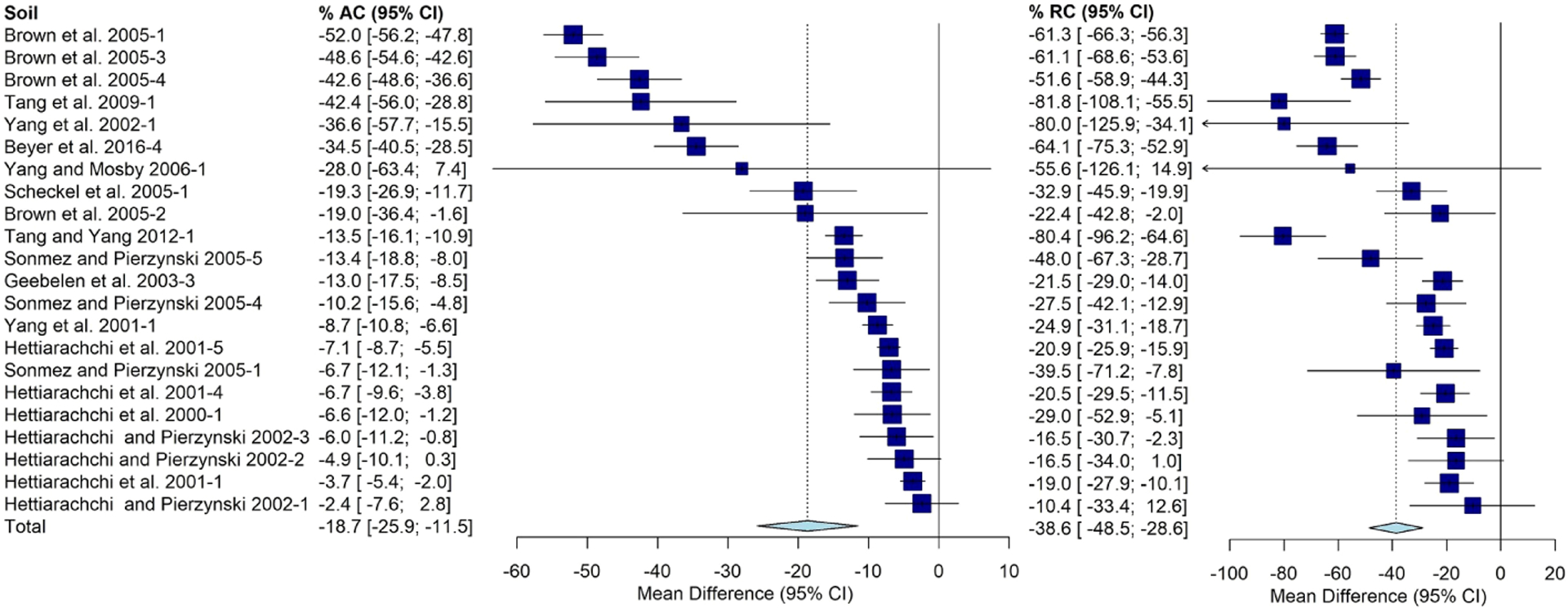
Random effects forest plot of AC and RC for soils from Jasper County, MO. Squares represent the mean difference for a soil and error bars are 95% CI. Diamonds represent the 95% CI for summary treatment effects.

Another complication was different studies investigating soils from the same or similar locations. Even if it was apparent that a soil was present in more than one published study, it was counted separately. This was most obvious with Jasper County Superfund site. Of the approximately 80 soils present in the primary and secondary groups, 22 soils investigated are from Jasper County, MO. There is a wide range of effects observed within these soils with a mean AC of −19% [−26, −11%] and mean RC of −39%[−49%, −29%] (Figure 7).

It is evident that Jasper County, MO is overrepresented in this analysis, but it is unclear how this may have affected the results.

### 4.5 Recommendations

Research continues on the effectiveness of soil amendments to reduce the bioaccessibility of toxic compounds. A set of minimum reported data would allow better comparison across studies and greatly improve the ability of future researchers to conduct meta-analysis. This should include the total amount of contaminant, clearly stating the pH and reagents of the *in vitro* bioaccessibility extraction, IVBA of the contaminant in the control, amount of the active amendment added on an mg kg^−1^ basis, and standard deviations/errors for control and treated materials. Soil properties should be reported including the soil pH (after incubation), clay content, organic C content, and iron and aluminum oxide content.

To best facilitate comparisons across future studies, researchers need to use more consistent methodology. As noted above, the analysis of the primary group had to assume equivalence between 9 different pH/*in vitro* bioaccessibility extraction method combinations, without the inclusion of EPA Method 1340. It is highly desirable for researchers to have an *in vitro* bioaccessibility method to evaluate the ability of P soil amendments to reduce IVBA Pb. Based on *in vivo* data, Ryan et al. (62) concluded EPA Method 1340 did not accurately predict the bioavailability of Pb in phosphate amended soils. No reduction in IVBA Pb was observed using EPA Method 1340 while a 71% reduction in bioavailable Pb was observed in a juvenile swine model accepted by U.S. EPA for determining RBA Pb in soil and solid wastes (63, 64). Studies investigating the modification of EPA Method 1340 from pH 1.5 to between pH 2 and 2.5 observed greater reductions in IVBA Pb due to the addition of P amendments at higher extraction pHs (44, 49, 56). This study found similar results; the primary and secondary groups, which include all IVBA extraction methods except EPA Method 1340, had an average RC close to −25% and EPA Method 1340 group had an average RC of −8%. As a temporary solution, we propose analyzing Pb contaminated soils remediated with P amendments with both EPA Method 1340, as it currently has regulatory acceptance for unamended soils, and a modified EPA Method 1340 at pH of 2.5 to better assess the change in IVBA Pb. We suggest the use of EPA Method 1340 at pH 2.5, as it has been shown to be well correlated with *in vivo* data for unamended soils (12). Development and validation of a new *in vitro* method or validation of an existing method for determining bioaccessible Pb is needed for soils remediated with P amendments in order to accurately predict changes in bioavailability in phosphate-amended soils.

In addition to having consistency in how data are reported and generated, a shared data repository should be created for soil remediation data. The National Institutes of Health support a number of databases that could serve as a template, such as Chemical Effects in Biological Systems (65). This would allow information that authors did not report but may have generated, such as specific soil properties, to be available. Available *in vivo* data could also be included with the bioaccessibility data for a soil. Imputed standard deviations would not be needed, thus better representing true data. Authors would be able to identify if the soil being tested was a unique material or the same as previously published studies. A shared data repository would ideally solve the challenges regarding missing soil characterization data, the potential misrepresentation of others’ data, and the identification of identical soils.

## 5 Conclusion

There are 44 studies that have investigated the ability of P amendments to reduce bioaccessible Pb, representing over 100 soils. Some soils have shown increases in Pb bioaccessibility while others have shown reductions over 50%. On average, the addition of P amendments to Pb contaminated soil reduced the bioaccessibility of Pb by 12% when compared to total Pb and by 25% when compared to control IVBA Pb. Studies that investigated changes in bioaccessible Pb with EPA Method 1340 observed lower reductions in bioaccessible Pb. While the addition of P amendments consistently reduced bioaccessible Pb, it may not be sufficient for a site to be considered remediated.

Qualitative factors affecting reduction of bioaccessible Pb include the P amendment added and the source of the Pb contamination. Soluble amendments, especially phosphoric acid, show greater reductions in bioaccessible Pb. Contamination associated with urban Pb sources show lower reductions than other Pb sources. Greater control IVBA Pb was correlated with greater reductions in bioaccessible Pb when compared to total Pb. Continuous factors affecting reduction of bioaccessible Pb were incubated soil pH with lower soil pH being correlated with greater reductions in bioaccessible Pb. Based on this analysis, the use of P amendments to reduce bioaccessible Pb is most effective when phosphoric acid is applied, the soil is acidic after treatment, and the site is not contaminated due to urban Pb sources. Additional research needs to be conducted with urban contaminated soils as the results of this meta-analysis do not adequately explain the lesser reductions observed. Further primary research is also needed to elucidate the relationships between soil properties and the ability of P amendments to reduce bioaccessible Pb.

To improve comparisons across studies, researchers need to support a validated *in vitro* bioaccessibility extraction for phosphate amended soil. Further *in vivo in vitro* validation studies are needed for phosphate amended soils, examining a range of soil properties, Pb concentrations, and Pb sources. Until this occurs, we propose that researchers use EPA Method 1340 adjusted to pH 2.5 in addition to an unmodified EPA Method 1340 to assess changes in IVBA Pb in soils treated with P amendments. Reporting a minimum set of data regarding the soil(s) properties being investigated will also improve comparisons across studies.

## Supplementary Material

Supplementary Material

## Figures and Tables

**TABLE 1:** Studies included in the primary group with the number of soils, non-control treatments and total number of samples in each study.

Reference	Soils	Treatments	Samples
Basta et al. (36)*	1	1	6
Hettiarachchi et al. (37)*	5	5	84
Yang et al. (38)	1	3	12
Geebelen et al. (15)	10	1	60
Tang et al. (39)	1	8	27
Brown et al. (17)*	2	3	15
Codling (40)	2	1	12
Yoon et al. (41)	2	5	36
Cao et al. (18)	2	3	24
Kilgour et al. (42)	1	3	8
Zupančič et al. (43)	1	6	21
Beyer et al. (35)	1	1	18
Obrycki et al. (44)	2	2	84
Sanderson et al. (45)*	4	2	36
Obrycki et al. (16)*	1	1	6
Gu et al. (46)	2	6	56
Alasmary (47)	2	19	63

* Corresponding authors provided additional information.

**TABLE 2:** Summary of pooled treatment effect *via* random effects model for each analysis group.

Group	n	Absolute change					Relative change				
		I^2^	τ	p-value	Average	95% confidence interval	I^2^	τ^2^	p-value	Average	95% confidence interval
Primary	40	98.6%	12.2%	<0.05	−11.4%	[−15.4%,−7.4%]	95.7%	15.4%	<0.05	−21.4%	[−26.7%, −16.1%]
Secondary	36	96.7%	16.4%	<0.05	−15.6%	[−21.4%,−9.7%]	94.3%	22.4%	<0.05	−32.3%	[−40.5%, −24.2%]
Secondary (no field studies)	28	96.9%	15.6%	<0.05	−12.4%	[−18.7%, −6.1%]	94.7%	20.2%	<0.05	−27.2%	[−35.4%, −18.9%]
Secondary (only field studies)	8	88.4%	15.0%	<0.05	−28.5%	[−41.8%, −15.2%]	87.4%	22.9%	<0.05	−53.6%	[−74.4%,−32.9%]
EPA Method 1340	23	98.6%	11.4%	<0.05	−5.6%	[−10.7%,−0.6%]	98.0%	17.2%	<0.05	−8.3%	[−16.4%, −0.8%]

I^2^ is percentage of difference due real differences, and τ is the between study standard deviation. The value of τ is in the same units as the treatment effects.

## Data Availability

The raw data supporting the conclusions of this article will be made available by the authors, without undue reservation.
