# Supplementary material for: Using phosphate amendments to reduce bioaccessible Pb in contaminated soils: A meta-analysis
Source: Front Soil Sci. Author manuscript; Available in PMC 2023 Feb 1. (PMC9890325; doi:10.3389/fsoil.2022.1028328)
Supplement: Supplementary Material [file NIHMS1861454-supplement-Supplementary_Material.docx]

Supplementary Material

# Supplementary Figures

Table S1. Summary of references included in each analysis group

| Reference | Analysis group |
| --- | --- |
| Alasmary 2020 | Primary |
| Barth et al. 2005 | EPA Method 1340 |
| Basta et al. 2001 | Primary |
| Beyer et al. 2016 | Primary |
| Beyer et al. 2016 | Secondary |
| Beyer et al. 2016 | EPA Method 1340 |
| Bosso et al. 2008 | Excluded |
| Brown et al. 2005 | Primary |
| Brown et al. 2005 | Secondary |
| Brown et al. 2007 | Secondary |
| Cai et al. 2017 | Secondary |
| Cao et al. 2009 | Primary |
| Codling 2007 | Primary |
| Cui et al. 2010 | Secondary |
| Cui et al. 2017 | Secondary |
| Geebelen et al. 2003 | Primary |
| Gu et al. 2020 | Primary |
| He et al. 2013 | Secondary |
| Hettiarachchi and Pierzynski 2002 | Secondary |
| Hettiarachchi et al. 2000 | Secondary |
| Hettiarachchi et al. 2001 | Primary |
| Hettiarachchi et al. 2001 | Secondary |
| Juhasz et al. 2016 | Secondary |
| Juhasz et al. 2016 | EPA Method 1340 |
| Kastury et al. 2019a | EPA Method 1340 |
| Kastury et al. 2019b | EPA Method 1340 |
| Kilgour et al. 2008 | Primary |
| Li et al. 2017 | Secondary |
| Li et al. 2017 | EPA Method 1340 |
| Madrid et al. 2008 | EPA Method 1340 |
| Mele et al. 2015 | EPA Method 1340 |
| Moseley et al. 2008 | Secondary |
| Moseley et al. 2008 | EPA Method 1340 |
| Obrycki et al. 2016 | Primary |
| Obrycki et al. 2016 | EPA Method 1340 |
| Obrycki et al. 2017 | Primary |
| Park et al. 2011 | EPA Method 1340 |
| Rizwan et al. 2016 | EPA Method 1340 |
| Sanderson et al. 2016 | Primary |
| Scheckel et al. 2005 | Secondary |
| Scheckel et al. 2005 | EPA Method 1340 |
| Schwab et al. 2006 | EPA Method 1340 |
| Seshadri et al. 2017 | EPA Method 1340 |
| Sharma et al. 2011 | Secondary |
| Sonmez and Pierzynski 2005 | Secondary |
| Tang and Yang 2012 | Secondary |
| Tang et al. 2009 | Secondary |
| Tang et al. 2004 | Primary |
| Yang and Mosby 2006 | Secondary |
| Yang et al. 2001 | Primary |
| Yang et al. 2002 | Secondary |
| Yoon et al. 2007 | Primary |
| Zupančič et al. 2012 | Primary |

References only in Supplementary Material

Barth, E. F., Succop, P. A., & Evans, M. L. (2005). Evaluation of lead availability in amended soils monitored over a long-term time period. *Environmental Monitoring and Assessment*, *110*(1–3), 257–270. <https://doi.org/10.1007/s10661-005-7696-5>

Bosso, S. T., Enzweiler, J., & Angélica, R. S. (2008). Lead bioaccessibility in soil and mine wastes after immobilization with phosphate. *Water, Air, and Soil Pollution*, *195*(1–4), 257–273. https://doi.org/10.1007/s11270-008-9744-6

Brown, S. L., Compton, H., & Basta, N. T. (2007). Field Test of In Situ Soil Amendments at the Tar Creek National Priorities List Superfund Site. *Journal of Environmental Quality*, *36*(6), 1627–1634. <https://doi.org/10.2134/jeq2007.0018>

Cai, M., McBride, M. B., Li, K., & Li, Z. (2017). Bioaccessibility of As and Pb in orchard and urban soils amended with phosphate, Fe oxide and organic matter. *Chemosphere*, *173*, 153–159. https://doi.org/10.1016/j.chemosphere.2017.01.049

Cui, Y., Du, X., Weng, L., & van Riemsdijk, W. H. (2010). Assessment of in situ immobilization of lead (Pb) and arsenic (As) in contaminated soils with phosphate and iron: Solubility and bioaccessibility. *Water, Air, and Soil Pollution*, *213*(1–4), 95–104. https://doi.org/10.1007/S11270-010-0370-8

Cui, H., Yang, X., Xu, L., Fan, Y., Yi, Q., Li, R., & Zhou, J. (2017). Effects of goethite on the fractions of Cu, Cd, Pb, P and soil enzyme activity with hydroxyapatite in heavy metal-contaminated soil. *RSC Advances*, *7*(72), 45869–45877. <https://doi.org/10.1039/C7RA08786A>

Hettiarachchi, G. M., Pierzynski, G. M., & Ransom, M. D. (2000). In situ stabilization of soil lead using phosphorus and manganese oxide. *Environmental Science and Technology*, *34*(21), 4614–4619. https://doi.org/10.1021/es001228p

Hettiarachchi, G. M., & Pierzynski, G. M. (2002). In situ stabilization of soil lead using phosphorus and manganese oxide: Influence of plant growth. *Journal of Environmental Quality*, *31*(2), 564–572. <https://doi.org/10.2134/jeq2002.5640>

Kastury, F., Placitu, S., Boland, J., Karna, R. R., Scheckel, K. G., Smith, E., & Juhasz, A. L. (2019). Relationship between Pb relative bioavailability and bioaccessibility in phosphate amended soil: Uncertainty associated with predicting Pb immobilization efficacy using in vitro assays. *Environment International*, *131*. <https://doi.org/10.1016/j.envint.2019.104967>

Kastury, F., Smith, E., Doelsch, E., Lombi, E., Donnelley, M., Cmielewski, P. L., Parsons, D. W., Scheckel, K. G., Paterson, D., de Jonge, M. D., Herde, C., & Juhasz, A. L. (2019). In Vitro, in Vivo, and Spectroscopic Assessment of Lead Exposure Reduction via Ingestion and Inhalation Pathways Using Phosphate and Iron Amendments. *Environmental Science & Technology*, *53*(17), 10329. https://doi.org/10.1021/ACS.EST.9B02448

Li, S.W., Liu, X., Sun, H.J., Li, M.Y., Zhao, D., Luo, J., Li, H.B., & Ma, L. Q. (2017). Effect of phosphate amendment on relative bioavailability and bioaccessibility of lead and arsenic in contaminated soils. *Journal of Hazardous Materials*, *339*, 256–263. <https://doi.org/10.1016/j.jhazmat.2017.06.040>

Madrid, F., Díaz-Barrientos, E., Florido, M. C., & Madrid, L. (2008). Inorganic amendments to decrease metal availability in soils of recreational urban areas: Limitations to their efficiency and possible drawbacks. *Water, Air, and Soil Pollution*, *192*(1–4), 117–125. <https://doi.org/10.1007/s11270-008-9639-6>

Mele, E., Donner, E., Juhasz, A. L., Brunetti, G., Smith, E., Betts, A. R., Castaldi, P., Deiana, S., Scheckel, K. G., & Lombi, E. (2015). In Situ Fixation of Metal(loid)s in Contaminated Soils: A Comparison of Conventional, Opportunistic, and Engineered Soil Amendments. *Environmental Science and Technology*, *49*(22), 13501–13509. <https://pubs.acs.org/doi/10.1021/acs.est.5b01356>

Park, J. H., Bolan, N. S., Chung, J. W., Naidu, R., & Megharaj, M. (2011). Environmental monitoring of the role of phosphate compounds in enhancing immobilization and reducing bioavailability of lead in contaminated soils. *Journal of Environmental Monitoring*, *13*(8), 2234–2242. https://doi.org/10.1039/c1em10275c

Rizwan, M. S., Imtiaz, M., Huang, G., Chhajro, M. A., Liu, Y., Fu, Q., Zhu, J., Ashraf, M., Zafar, M., Bashir, S., & Hu, H. (2016). Immobilization of Pb and Cu in polluted soil by superphosphate, multi-walled carbon nanotube, rice straw and its derived biochar. *Environmental Science and Pollution Research*, *23*(15), 15532–15543. <https://doi.org/10.1007/s11356-016-6695-0>

Schwab, A. P., Lewis, K., & Banks, M. K. (2006). Lead stabilization by phosphate amendments in soil impacted by paint residue. *Journal of Environmental Science and Health - Part A Toxic/Hazardous Substances and Environmental Engineering*, *41*(3), 359–368. <https://doi.org/10.1080/10934520500423493>

Seshadri, B., Bolan, N. S., Choppala, G., Kunhikrishnan, A., Sanderson, P., Wang, H., Currie, L. D., Tsang, D. C. W., Ok, Y. S., & Kim, K. (2017). Potential value of phosphate compounds in enhancing immobilization and reducing bioavailability of mixed heavy metal contaminants in shooting range soil. *Chemosphere*, *184*, 197–206. <https://doi.org/10.1016/j.chemosphere.2017.05.172>

Sharma, M. C., Saxena, R., Sharma, S. K., & Singh, S. (2011). Modelling of Heavy Metal Mobility in Delhi Soils before and after Remediation with Green Amendment Rock Phosphate using Sequential Extraction, TCLP and PBET. *Asian Journal of Water, Environment and Pollution*, *8*, 25–33.

Sonmez, O., & Pierzynski, G. M. (2005). Phosphorus and manganese oxides effects on soil lead bioaccessibility: PBET and TCLP. *Water, Air, and Soil Pollution*, *166*(1–4), 3–16. https://doi.org/10.1007/s11270-005-8088-8
